# Minimum effective local anesthetic volume for surgical anesthesia by subparaneural, ultrasound-guided popliteal sciatic nerve block: A prospective dose-finding study

**DOI:** 10.1097/MD.0000000000004652

**Published:** 2016-08-26

**Authors:** Seung Uk Bang, Dong Ju Kim, Jin Ho Bae, Kyudon Chung, Yeesuk Kim

**Affiliations:** a Department of Anesthesiology and Pain Medicine, College of Medicine, the Catholic University of Korea, Seoul, Republic of Korea; b Department of Surgery, College of Medicine, Chungbuk National University, Cheongju, Republic of Korea; c Department of Anesthesiology and Pain Medicine, College of Medicine, Chungbuk National University, Cheongju, Republic of Korea.

**Keywords:** Effective dose, Minimal effective volume, Nerve block, Ropivacaine, Sciatic nerve, Surgical anesthesia, Ultrasound

## Abstract

Because of its rapid onset time, recent years have seen an increase in the use of ultrasound (US)-guided popliteal sciatic nerve block (PSNB) via subparaneural injection for induction of surgical anesthesia. Moreover, in below-knee surgery, combined blocks, as opposed to sciatic nerve block alone, have become more common. These combined blocks often require a large volume of local anesthetic (LA), thus increasing the risk of local-anesthetic systemic toxicity (LAST). Thus, to decrease the risk of LAST, it is important to know the minimum effective volume (MEV) required for an adequate block. We, therefore, aimed to determine the MEV of ropivacaine 0.75% for induction of surgical anesthesia by the method of US-guided popliteal sciatic nerve block via subparaneural injection.

Thirty patients underwent a US-guided PSNB with ropivacaine 0.75% at a 20-mL starting volume. Using a step-up/step-down method, we determined injection volumes for consecutive patients from the preceding patient's outcome. When an effective block was achieved within 40 minutes after injection, the next patient's volume was decreased by 2 mL. If the block failed, the next patient's volume was increased by 2 mL. The sensory and motor blockade was graded according to a 4-point scale. The block was considered a success if a combination of anesthesia and paresis (a score of 3 for both the sensory and motor nerves) was achieved within 40 minutes. The primary outcome measure was the MEV resulting in a successful subparaneural block of the sciatic nerve in 50% of patients (MEV_50_). Additionally, the data were processed with a probit regression analysis to determine the volume required to produce a complete sciatic nerve block in 90% of subjects (ED_90_).

The MEV_50_ of 0.75% ropivacaine is 6.14 mL (95% confidence interval, 4.33–7.94 mL). The ED_90_ by probit analysis for a subparaneural injection was 8.9 mL (95% CI, 7.09–21.75 mL).

The 6.14-mL MEV_50_ of ropivacaine 0.75% represents a 71% reduction in volume compared with neurostimulation techniques and a 14.7% reduction in volume compared with US-guided PSNB using the alternative perineural injection technique.

## 1 Introduction

Sciatic nerve blocks are used extensively for surgical anesthesia as well as for postoperative analgesia in below-knee surgery. A popliteal sciatic nerve block (PSNB) is most commonly used because the sciatic nerve is located superficially and is relatively easy to identify when the popliteal artery is used as an anatomical landmark. Moreover, most proximal approaches above the mid-femoral area result in a similar extent of sensory blockade, since, except for the parasacral sciatic nerve block, they do not block the posterior femoral cutaneous nerve. At the same time, they lead to an unwanted motor block in the hamstring muscle, which causes weakness in knee flexion.^[1,2]^

Recent studies have found a “sweet spot” on the sciatic nerve in ultrasound (US)-guided PSNB, and there are several advantages over a conventional perineural technique in using this “sweet spot.” Since the existence of a common epineural sheath was first reported in an anatomical study, there have been several studies of the paraneural sheath, which has been variously called the popliteal nerve sheath, the sciatic fascial sheath, the common epineural sheath, and the complex fascial layer.^[3–6]^

One study named the space between the paraneural sheath and the epimysium of the adjacent thigh muscle as the subepimyseal space, and the space between the paraneural sheath and the epineurium as the subparaneural space, which act as conduits for local anesthetic (LA) spread during a sciatic nerve block.^[6]^ It has been reported that injection into the subparaneural space has several advantages over conventional perineural injection. A subepineural (subparaneural) injection required shorter performance and onset times than separate injections around the tibial and common peroneal nerves and resulted in a higher incidence of complete sensory blockade.^[7]^ Another study reported that this technique improved the quality of the sensory block and resulted in a longer block duration. Because of the advantages of subparaneural injection, it is becoming more frequently used in US-guided PSNB for surgical anesthesia.

In addition, in US-guided PSNB for surgical anesthesia, there has been an increase in the use of combined blocks rather than just sciatic nerve block alone, which include femoral and saphenous nerve blocks. However, a large volume of LA is needed for such combined blocks, thus increasing the risk of local-anesthetic systemic toxicity (LAST).^[8]^ Therefore, to decrease the risk of LAST, it is important to know the minimum effective volume (MEV) and effective dose (ED) required for an adequate block. There have been a few studies on effective volumes for US-guided PSNB via the *perineural* route.^[9–11]^ However, as far as we know, no previous study has sought to determine the minimum effective volume for surgical anesthesia via the *subparaneural* route. The purpose of the present study was to determine the MEV_50_ and ED_90_ for surgical anesthesia in US-guided PSNB via subparaneural injection.

## 2 Methods

This study has been approved by the Catholic University Hospital Institutional Review Board, Daejeon, Korea (DC13OISI00036) and registered with the WHO Clinical Research Information Service (http://cris.nih.go.kr/cris, KCT0001643). Written, informed consent was obtained from all patients enrolled in the study. Alternative anesthetic practices that are standard in our hospital for below-knee surgery comprise central neuraxial block, general anesthesia, and peripheral nerve block (PNB). Patients were informed about the different types of anesthesia that could be used for surgery, and if they agreed to take part in the study and receive PNB, they were informed about the risk of conversion to general anesthesia if the sciatic nerve block failed.

Thirty patients aged 19 to 70 years and scheduled for elective below-knee surgery under a PNB were included. Their physical status was rated I to II by the criteria of the American Society of Anesthesiologists (ASA). Exclusion criteria were clinically significant coagulopathy, infection at the injection site, allergy to the LA, severe cardiopulmonary disease (ASA ≥III), a body mass index >35 kg/m^2^, diabetic or other neuropathy, prior surgery in the popliteal region, or receiving opioids for chronic analgesic therapy.

At our institution, we routinely administer PNBs for surgical anesthesia in a separate “block room” furnished with monitoring devices, drugs, an emergency kit, and a LAST kit. All the patients in this study received PSNB in this room. These procedures were performed 1 hour early, with the consent of patients and surgeon, because of the long preparation time and the prolonged onset time. As preferred by both the surgeon and patients, we did not use a thigh tourniquet. All procedures were performed under ultrasonographic guidance (WS80A, Samsung Medicine, Seoul, Korea).

Eighteen-gauge intravenous access was established in the forearm in the general ward. After arrival in the block room, we applied supplemental oxygen by facial mask at 5 L/min and standard monitoring (noninvasive arterial blood pressure, electrocardiography, and pulse oximetry) throughout anesthesia. Patients were premedicated with intravenous midazolam (0.02 mg/kg) and fentanyl (0.5 μg/kg). All blocks were conducted by one anesthesiologist (author S.B.) who had substantial expertise in US-guided PNBs. The US-guided PSNBs of this study were performed using a subparaneural injection technique. When the surgical procedure involved the medial aspect of the calf and ankle, an additional US-guided femoral nerve block was performed.

### 2.1 Nerve block technique

With the patient in the supine position, the femoral nerve was blocked with sterile technique using a linear probe at the inguinal crease. After confirming the femoral vessels, fascia iliaca, and femoral nerve, a 22G Tuohy needle (Contiplex Tuohy Set, B. Braun Melsungen AG, Melsungen, Germany) was inserted in-plane in a lateral-to-medial orientation and advanced toward the fascia iliaca and femoral nerve. After confirming the passage of the needle through the fascia iliaca using fascial click and a small amount of saline, we injected 10 mL of mepivacaine 1.5% with epinephrine 5 μg/mL between the fascia iliaca and the femoral nerve.

**Figure 1:**
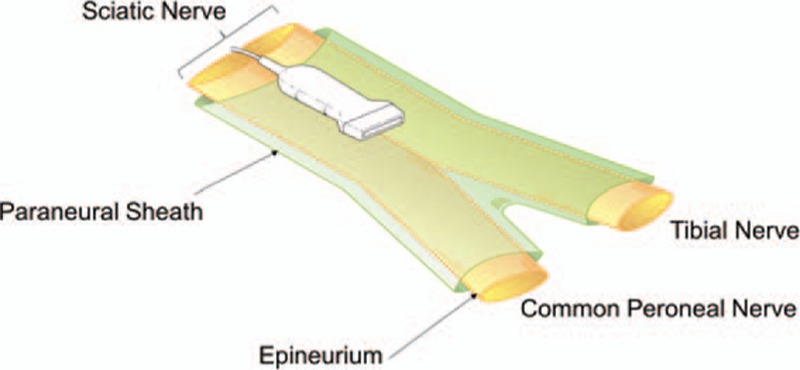
Schematic diagram illustrating the anatomy of the paraneural sheath and the sciatic nerve at the popliteal fossa. The sciatic nerve is surrounded by the paraneural sheath. The subparaneural space is the space inside the paraneural sheath.

**Figure 2:**
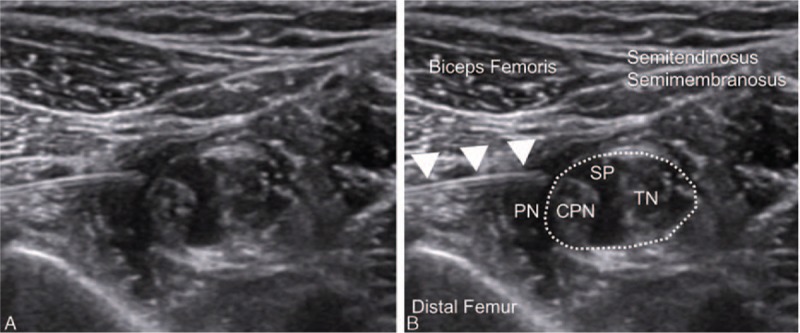
Ultrasound image of the perineural and subparaneural spaces. Shown are the paraneural sheath surrounding the sciatic nerve and the perineural space between the epimysium of the adjacent muscles and the paraneural sheath. The subparaneural space is located inside the paraneural sheath (dotted line). CPN = common peroneal nerve, PN = perineural space, SP = subparaneural space, TN = tibial nerve.

After turning the patient from a supine to a non-dependent lateral position in bed, the skin was prepared with povidone-iodine and chlorhexidine solutions. The ultrasound probe was placed transversely across the popliteal fossa at the popliteal crease. After confirming the popliteal artery, vein, and tibial nerve, the probe was moved proximally to find the bifurcation of the sciatic nerve. The neural bifurcation was identified as the point where both branches are contiguous and display a bilobular pattern. The insertion point of the needle was determined as the point of minimum distance measuring from the skin to the upper margin of the sciatic nerve. A skin wheal was raised with 1 mL of mepivacaine 1.5%. Using an in-plane technique, a 22G Tuohy needle was advanced in parallel with the linear probe until the tip reached the epimysium of adjacent muscle around the sciatic nerve. After advancing inside the epimysium under real-time ultrasonic guidance, we widened the subepimyseal perineural space (perineural space) between the epimysium and the paraneural sheath using saline (0.5–1 mL). After identifying the paraneural sheath clearly, the needle was advanced within the paraneural sheath (Figs. 1 and 2). To confirm accurate needle tip positioning beneath the paraneural sheath, we used two confirmatory steps: detecting a tactile, fascial “click” on needle passage through the paraneural sheath and observing immediate separation of the adjacent tibial nerve and common peroneal nerve upon injection of saline (0.5 mL) without neural swelling. At this point, a previously determined volume of ropivacaine 0.75% (AstraZeneca, Luton, UK) was injected slowly after negative blood aspiration. If neural swelling was detected under ultrasound, the needle was carefully withdrawn before resuming the injection. After LA injection, the 22G Tuohy needle was removed. An 18-gauge, 10-cm Tuohy needle was then advanced at the same insertion site to the perineural space (not the subparaneural space). Then, a 19-gauge catheter was advanced under direct vision 1 cm past the needle tip in the perineural space. The purpose of the catheter was to allow LA re-injection for additional complete anesthesia and postoperative analgesia in the patient with incomplete block.

After LA injection, the sensory and motor blocks were evaluated every 5 minutes for 40 minutes. When an effective sciatic nerve block was achieved within 40 minutes after injection, the injection volume for the next patient was decreased by 2 mL (10% of the initial volume). Conversely, if the block failed, the next patient's volume was increased by 2 mL. The maximum dose used in this study was limited to 20 mL of ropivacaine 0.75% to prevent LA toxicity. The study plan also called for stopping after obtaining 5 consecutive successful blocks requiring less than 1 mL each, because this study was not designed to measure such small quantities.

### 2.2 Nerve block assessment

After LA injection, the sensory and motor block were evaluated every 5 minutes for 40 minutes by an independent observer (author K.C.), who was not present during block administration and was blinded to the injected volume. Using a 23-gauge needle and cold ice, the observer assessed the sensory block in the distributions of the tibial nerve (plantar surface of the foot), deep peroneal nerve (first and second web spaces of the toes), and superficial peroneal nerve (dorsal surface of the foot). Additionally, the presence of motor blockade was tested in the tibial nerve (plantar flexion of the foot) and common peroneal nerve (dorsiflexion of the foot). The sensory and motor blockades were graded on a 4-point scale (Table 1).

**Table 1:**
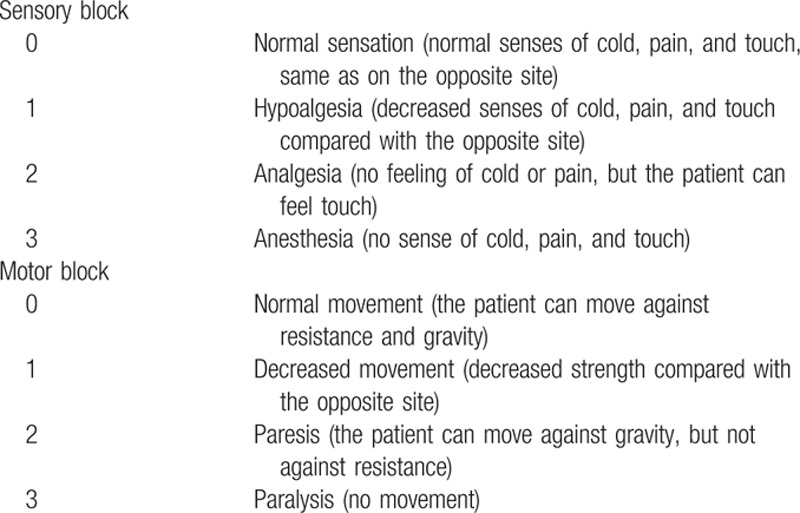
Assessments of sensory and motor blocks.

The block was considered a success if anesthesia and paresis were achieved within 40 minutes (a score of 3 for both sensory and motor nerves). If the blockade failed, it was classified as an incomplete block of the sensory and/or motor nerves. In patients with an ineffective sensory block, an additional bolus of ropivacaine 0.75% was reinjected through the perineural catheter. The additional volume was determined as the volume obtained by subtracting the amount that had already been injected from 20 mL of ropivacaine. Nevertheless, if this did not produce anesthesia adequate for the completion of surgery within 10 minutes of re-injecting LA, patients underwent general anesthesia via the supraglottic airway. Patients with only an incomplete motor block proceeded to surgery because a complete sensory block was sufficient for this. Propofol infusion was titrated to light sleep equivalent to 5 on the Ramsey scale, at which patients exhibit a sluggish response to a light glabellar tap or to a loud auditory stimulus during surgery.^[12]^

All patients received intravenous ketorolac 30 mg at the end of surgery. Postoperative pain was assessed in the post-anesthesia care unit (PACU) using a visual analog score (VAS), in which a score of 0 indicated no pain and a score of 10 indicated the most severe pain. If the VAS was more than 4 points in the PACU, intravenous tramadol 25 mg was to be prescribed as rescue analgesia. Postoperative analgesia consisted of oral celecoxib 200 mg twice daily for 72 hours after surgery. In addition, intravenous fentanyl infusion was to be started in the PACU using a patient-controlled analgesia (PCA) pump. The PCA was programmed to deliver a bolus dose of 0.5 μg/kg, without background infusion, with a lockout of 7 minutes, and a 4-hour limit of 4 μg/kg.

The primary outcome measure was the MEV resulting in a successful subparaneural block of the sciatic nerve in 50% of patients (MEV_50_). The ED_90_ was calculated using a probit regression analysis. During the injections, any vascular punctures, paresthesias, neural swellings, or other complications were to be recorded. Three days after surgery, all patients were further interviewed by an investigator blinded to the patient doses to inquire about complications, such as persistent paresthesia and motor deficits in the block-related area. Finally, all patients were booked for a visit to the outpatient department 4 weeks postoperation for follow up by an orthopedic surgeon. The following block-related data were defined: imaging time (the time interval between contact of the US probe with the popliteal crease and the acquisition of a satisfactory picture), the needling time (the time interval between raising the skin wheal and the completion of LA injection), and the performance time (the sum of the imaging and needling times).

### 2.3 Statistical analysis

The primary outcome variable was the MEV of ropivacaine 0.75% providing sensory and motor blocks of the sciatic nerve adequate for surgical anesthesia in 50% of patients (MEV_50_). The MEV_50_ of 0.75% ropivacaine was defined as the midpoint of pairs of volumes from consecutive patients in which a negative response (an incomplete block within 40 minutes) was followed by a positive response (a complete block within 40 minutes). To calculate the MEV_50_ of ropivacaine, 0.75% with the sequential up-and-down technique, we determined *a priori* that a minimum of 5 independent negative-positive, up-and-down deflections must be observed during data collection.^[13,14]^ The data were also processed with a probit regression analysis to determine the volume of 0.75% ropivacaine required to produce within 40 minutes of injection a complete sciatic nerve block in 90% (ED_90_) of the subjects. For continuous data, normality was first assessed with the Kolmogorov–Smirnov test. Normal data were then analyzed using Student *t* test with unequal variances. Data that did not have a normal distribution were analyzed with the Mann–Whitney *U* test. Continuous variables are presented as the mean with standard deviation (SD) or 95% confidence interval (CI) in parentheses, or as the median with range in parentheses, according to the data distribution. The categorical variables are presented as a number with a percentage in parentheses. Summary statistics were calculated using SPSS 20.0 statistical software for Windows (SPSS Inc., Chicago, IL, USA). A value of *P* < 0.05 was considered significant.

## 3 Results

**Figure 3:**
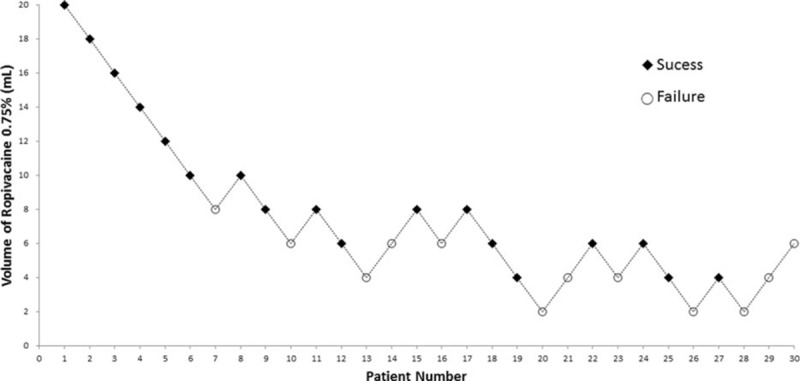
The up-and-down sequence of volumes of ropivacaine 0.75% required to produce surgical anesthesia. Squares represent successful blocks and circles represent failed blocks.

Patient characteristics and anesthetic-block-related data are presented in Tables 2 and 3. Thirty patients were enrolled in this study. Twenty-three patients received only PSNB, and 7 patients received combined PSNB and femoral nerve block. The subject data were analyzed to evaluate the MEV of LA required for a subparaneural PSNB for surgical anesthesia. The sequence of positive and negative responses recorded in consecutive patients is shown in Fig. 3.

**Table 2:**
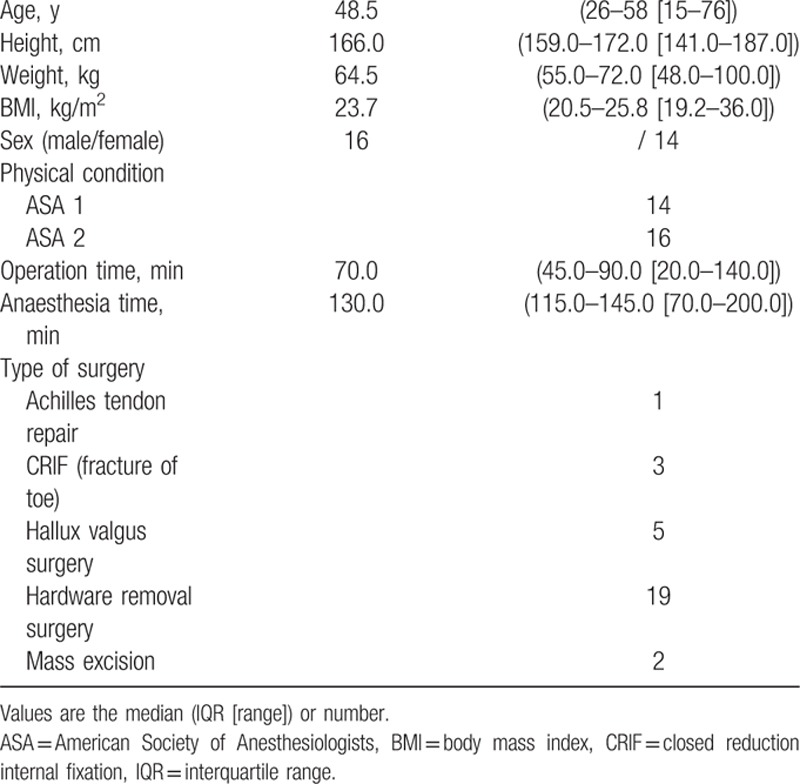
Patient characteristics.

**Table 3:**
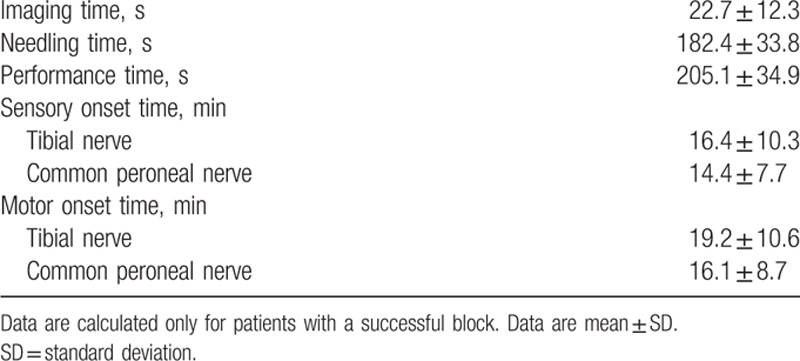
Block-related data.

The volume of ropivacaine 0.75% resulting in a successful subparaneural block of the sciatic nerve in 50% of patients (MEV_50_), according to the up-and-down sequence, was 6.14 mL (95% CI, 4.33–7.94 mL). The ED_90_ by probit analysis for a subparaneural injection providing an adequate sciatic nerve block was 8.9 mL (95% CI, 7.09–21.75 mL).

In the 12 patients with block failures, 5 had only an incomplete motor block, and 7 had both incomplete sensory and incomplete motor blocks. The patients who had only incomplete motor blocks proceeded with the operation and the 7 patients with incomplete motor and sensory blocks were administered an additional bolus of ropivacaine 0.75% through the perineural catheter. Among these, 3 patients achieved sensory block and proceeded with the surgery and 4 patients were placed under general anesthesia. During the sciatic nerve block, no paresthesias or vascular punctures were noted. No patient reported a VAS of more than 4 points or needed additional tramadol for analgesia in the PACU. The follow-ups of all patients at 3 days and 4 weeks postoperation were uneventful.

## 4 Discussion

The objective of this study was to determine the MEV_50_ and ED_90_ for surgical anesthesia in a US-guided PSNB where subparaneural technique is used. We found that the MEV_50_ was 6.14 mL and the ED_90_, as estimated by probit regression analysis, was 8.9 mL.

Taboada et al^[15]^ have reported an MEV_50_ of 20 ± 3 mL in PSNB using neurostimulation technique. PSNB using US-guided technique, perineural injection of 0.5% ropivacaine, the up-and-down method, and isotonic regression^[10]^ found ED_50_ and ED_95_ volumes of 6 and 16 mL, respectively. The volumes used in the latter study were 47% less than in the study with neurostimulation technique owing to the contribution of ultrasonic guidance.

For comparison, we converted the data from the above 2 studies into MEV_50_ figures using the same algorithm as we use here for our own data. The MEV_50_s were 21.8 mL (95% CI, 18.9–24.6 mL) in neurostimulation and 7.2 mL (95% CI, 4.09–10.31 mL) in US-guided perineural PSNB. Our study yielded an MEV_50_ of 6.14 mL, which is a 71.8% reduction compared with neurostimulation technique and a 14.7% reduction compared with a perineural injection technique.

We consider the reason for this to be LA spread within a more compact space during a subparaneural injection compared with a perineural injection. The sciatic nerve is surrounded by a fascia (paraneural sheath), which is a structure distinct from the epineurium and has distinct gross anatomic, histologic, and sonographic features. LA spread along the nerve was reported to be more extensive and of greater distance when injected into the space under the fascia (subparaneural injection), while perineural injection spreads diffusely into the fatty tissue, is less extensive, and remains confined to the site of injection.^[5]^ Moreover, subparaneural injection involves trapping the ropivacaine under the paraneural sheath and thus increasing the exposure of the nerve to ropivacaine molecules. Moreover, subparaneural injection readily achieves double circumferential spread around the tibial and peroneal nerves.^[7,16]^

A single MEV study of US-guided PSNB for analgesia using the subparaneural injection technique has recently been reported.^[17]^ A combination of lidocaine 1.0%, bupivacaine 0.25%, and epinephrine 5 μg/mL was injected at the neural bifurcation. A volume assignment was carried out using a sequential up-and-down method, where the volume of LA administered to each patient depended on the response of the previous one. Using isotonic regression and a bootstrap confidence interval, the MEV_90_ was estimated as 13.3 mL (95% CI, 10.2–16.4 mL). This is very valuable information, explaining why only one such study has so far appeared. However, success was evaluated with a composite sensorimotor score (sensory block: 0 = no block, 1 = analgesia, 2 = anesthesia; motor block: 0 = no block, 1 = paresis, 2 = paralysis), and the block was considered a success if a minimum composite score of 6 points out of 8 was achieved within 30 minutes of LA injection. Although the blockade of the sensory nerve was incomplete, the nerve block was considered a success if it attained a score of >6. Accordingly, as the authors mention, the results of the study are relevant to postoperative analgesia, but not to surgical anesthesia.

On the other hand, the present study used grade scoring in which success depended on whether the blockade was complete for both the sensory and motor nerves. If either blockade was incomplete, it was not considered a success. That is, we report a volume effective for complete surgical anesthesia.

This study has some limitations. First, while a previous study evaluated success at 30 minutes after injection, our study evaluated the success of the sciatic nerve block at 40 minutes. Five (27%) out of our 18 patients with successful blocks were considered to have developed a successful block between 30 and 40 minutes. Moreover, other studies have obtained results similar to ours. A complete block has been observed for both sensory and motor nerves in 57% of patients at 30 minutes after injection and in 88% of patients on arrival in the recovery room.^[16]^ This demonstrates that the choice of post-injection evaluation time influences the apparent success of the block. However, in general, the evaluation times of previous studies have been diverse (e.g., 30, 45, and 60 minutes) because the onset time of sciatic nerve block using ultrasound is 30 to 60 minutes.^[9–11,17]^

A second limitation is that pressure monitoring during injection, generally thought to decrease neurologic complications and to produce a constant rate of injection, was not used, because the variable we needed to control was flow rate. No capability for flow rate monitoring during injection is yet available commercially, including from the BSmart^TM^ Injection Pressure Monitor (B. Braun Melsungen AG, Melsungen, Germany).^[18,19]^ Although we tried to ensure that the injection speed was controlled, we cannot be sure of this. Moreover, we cannot exclude the possibility that a relatively rapid injection produces a wider spread of ropivacaine along the sciatic nerve.

A third limitation is that ropivacaine was used here at a standard concentration of 0.75% to block the sciatic nerve; the concentration variable was not explored. Although it is possible to block only the sciatic nerve, most surgeries require multiple peripheral nerve blocks for anesthesia, which may unacceptably increase the risk of LAST even with the MEV determined here. Further studies are needed to decrease the risk of LAST in these cases, and should determine the MEV at lower concentrations and the minimum effective concentration.

In conclusion, the present study documents the MEV of ropivacaine 0.75% required for surgical anesthesia in a US-guided PSNB using a subparaneural injection technique. The MEV_50_ of ropivacaine 0.75% was 6.14 mL (95% CI, 4.33–7.94 mL). The ED_90_ was estimated with a probit regression analysis as 8.9 mL. The MEV_50_ of 6.14 mL found here is a 71% reduction in volume compared with neurostimulation techniques, and a 14.7% reduction in volume compared with US-guided PSNB using a perineural injection technique.
